# Real‐world treatment patterns, adverse events and clinical outcomes in patients with chronic lymphocytic leukaemia treated with ibrutinib in the UK

**DOI:** 10.1002/jha2.174

**Published:** 2021-03-13

**Authors:** Peter Hillmen, Jing Xie, Alan S. M. Yong, Catherine Waweru, Thuy Anh Sorof, Ravi K. Goyal, Keith L. Davis

**Affiliations:** ^1^ St James's University Hospital Leeds UK; ^2^ AstraZeneca Gaithersburg Maryland USA; ^3^ AstraZeneca Luton UK; ^4^ Acerta Pharma (a member of the AstraZeneca group) South San Francisco California USA; ^5^ RTI Health Solutions Research Triangle Park North Carolina USA

**Keywords:** Bruton tyrosine kinase, chronic lymphocytic leukaemia, ibrutinib, tyrosine kinase inhibitor

## Abstract

Chronic lymphocytic leukaemia (CLL) is the most common leukaemia in adults in the UK. Ibrutinib, an oral Bruton tyrosine kinase inhibitor (BTKi) for CLL approved by the UK's National Institute for Health and Care Excellence in January 2017, represented a major shift in CLL management. Real‐world data on ibrutinib use in routine clinical practice will inform patients’ and physicians’ decision‐making. We conducted a retrospective medical chart review of UK patients with CLL who initiated ibrutinib between January 2017 and July 2018. Data for 259 patients were contributed by 34 haematology‐oncology specialists, with a median follow‐up duration of 16.7 months. Median age at ibrutinib initiation was 71 years. Ibrutinib first‐line monotherapy was prescribed in 20.1% of patients. Ibrutinib was permanently discontinued in 15.4% of patients, mostly owing to progressive disease. Adverse events (AEs) were reported in 151 patients (58.3%). The most common were bruising (19.3% of patients), cytopenias (17.0%) and diarrhoea (11.6%). Ibrutinib dose reduction was observed in 14.3% of patients and was temporarily discontinued in 10.4% of patients, with the main reason for both being toxicity. These results expand the real‐world evidence on ibrutinib therapy and demonstrate a high burden of AEs, highlighting the need for more tolerable BTKis.

## 1 INTRODUCTION

Chronic lymphocytic leukaemia (CLL) is the most common leukaemia in adults in the UK [1], with approximately 3800 new cases diagnosed every year [2]. The median age at onset is about 70 years, and the median age at initiation of therapy is about 75 years; patients are often observed for several years before starting treatment [3].

Before the development of targeted oral therapies, systemic cytotoxic chemotherapies were the mainstay of CLL treatment, including chemoimmunotherapy with a combination of agents such as fludarabine, cyclophosphamide and rituximab (FCR) or bendamustine and rituximab (BR) [4]. However, the efficacy of these approaches is limited by toxicity, particularly in older patients [5].

Ibrutinib is a small‐molecule inhibitor of Bruton tyrosine kinase (BTK), a non‐receptor kinase that plays a critical role in the survival of leukaemic B cells in CLL [6, 7]. Originally approved for the treatment of mantle cell lymphoma, ibrutinib gained approval for use in CLL following positive results from the RESONATE phase 3 clinical trial, which demonstrated significant improvements in 12‐month overall survival (OS) and progression‐free survival (PFS) in patients with previously treated CLL receiving ibrutinib compared with ofatumumab [8]. In the subsequent RESONATE‐2 trial, ibrutinib had a significantly higher overall response rate and lower risk of progression or death than chlorambucil in treatment‐naive patients [9].

In January 2017, ibrutinib gained approval for reimbursement from the UK's National Institute for Health and Care Excellence (NICE) for use in patients with relapsed/refractory CLL, as well as for first‐line treatment of patients who have a 17p deletion or *TP53* mutation [10], with baseline funding from the Cancer Drugs Fund starting in April 2017 [11]. Ibrutinib treatment typically continues until disease progression or CLL transformation, although patients may also discontinue therapy owing to adverse events (AEs) [12]. Results from observational studies have shown an association between early discontinuation of ibrutinib and poor outcomes, such as reduced OS and excess mortality [13, 14].

Other targeted oral therapies with different mechanisms of action have also been approved for use in CLL. Idelalisib, an oral PI3K inhibitor, was approved by NICE for use in patients with CLL in combination with rituximab in 2015 [15], and venetoclax, an oral Bcl‐2 inhibitor, gained NICE approval for use in patients with CLL in October 2017 [16]. Given that targeted oral therapies have shifted the gold standard of CLL treatment away from systemic chemoimmunotherapy, patients and physicians now have several options to consider depending on individual circumstances.

While randomised clinical trials remain the gold standard for evidence‐based medicine, real‐world evidence can help to bridge knowledge gaps and to inform decision‐making by providing insights into the routine use of oral‐targeted therapies after approval for their use in CLL in the UK. Previous real‐world observational studies of ibrutinib in patients with CLL have been conducted in other countries [17, 18], have focused on a single centre [19] or have been limited to relapsed/refractory disease [14], and therefore do not provide a complete picture of ibrutinib treatment in the UK.

The present study was designed to investigate real‐world treatment patterns, clinical outcomes, incidence of AEs, time to and reasons for discontinuation and healthcare resources used for patients with CLL receiving treatment with ibrutinib in the UK. These study outcomes were also assessed among cohorts of patients with CLL who received treatment with venetoclax or idelalisib.

## 2 METHODS

This study was a retrospective, multicentre, observational, medical chart review carried out by eligible physicians in the UK.

### 2.1 Eligibility

Eligible physicians were haematology‐oncology specialists who had been practising in the UK for at least 3 years, had managed at least five patients with CLL in the past year and who spent at least 50% of their time in patient care. Patient inclusion and exclusion criteria are summarised in Table 1.

**TABLE 1:** Patient inclusion and exclusion criteria

**Inclusion criteria**	
Confirmed diagnosis of CLL ≥18 years of age at the time of CLL diagnosis Initiated treatment with ibrutinib, venetoclax or idelalisib after CLL diagnosis and between 31 January 2017 and 30 July 2018	

**Exclusion criteria**	
History of another malignancy before CLL diagnosis, except for basal cell or non‐metastatic squamous cell carcinoma of the skin, or carcinoma *in situ* of the cervix or breast Missing data on the date of ibrutinib, venetoclax or idelalisib initiation or date of death (or date of last available follow‐up for those alive at the time of data extraction) Enrolment in an international clinical trial for an experimental treatment related to CLL at any time History of Richter transformation before initiation of ibrutinib, venetoclax or idelalisib therapy (in respective cohorts)	

### 2.2 Data collection

Participating physicians reviewed medical records for eligible patients and entered information into a web‐based electronic data collection form. Baseline data were assessed at or within 12 months of the study index date, defined as the date of ibrutinib, venetoclax or idelalisib initiation during the case selection window. The case selection window began on 31 January 2017 for the ibrutinib cohort, 8 November 2017 for the venetoclax cohort and 28 October 2015 for the idelalisib cohort, and concluded on 30 July 2018 for all three cohorts.

Baseline data included patient demographics, performance status measured using the Eastern Cooperative Oncology Group (ECOG) performance status [20], comorbidities and Rai [21] and Binet [22] staging of CLL. High‐risk prognostic genetic factors were obtained where available.

Data on treatment characteristics included lines and regimens of CLL therapy, duration of therapy and time to and reasons for dose reductions and temporary discontinuation (defined as a treatment gap of longer than 14 days before re‐initiating therapy) or permanent discontinuation, identified based on the dates of beginning and end of therapy in patient records, along with other available notes. Overall response rates (complete response plus partial response) and clinical benefit rates (overall response plus stable disease) based on physician's (or designated clinical staff's) judgement were also collected. Safety data included incidence of AEs during treatment with the drug of interest.

Collected healthcare resource utilization data comprised the total number of healthcare visits during therapy and after discontinuation. Healthcare visits were classified as inpatient, outpatient, emergency department or general practitioner consultation. The number of patient visits to different healthcare services was standardised at the monthly level.

Survival outcomes included cause of death (CLL‐related or all‐cause), OS from treatment initiation and PFS (defined as the time from start of treatment to disease progression or death).

### 2.3 Statistical analyses

Kaplan‐Meier curves were used to estimate OS and PFS. Paired Wilcoxon signed‐rank tests were applied to assess differences between the number of patient healthcare visits per month during treatment and after discontinuation. All other data were summarised descriptively.

## 3 RESULTS

### 3.1 Patient demographics and baseline disease characteristics

Thirty‐four haematology‐oncology specialists contributed data for 259 patients with CLL who initiated treatment with ibrutinib between 31 January 2017 and 30 July 2018. The 259 patients in the ibrutinib cohort had a median age of 71 years at initiation of ibrutinib, and the majority were male (55.6%) and white (87.3%) (Table 2).

**TABLE 2:** Baseline demographics and clinical characteristics of patients in the ibrutinib cohort

**Measures**	**Ibrutinib cohort (*N* = 259)**
**Age at index date, years**	
Mean (SD)	71.3 (9.7)
Median	71
Minimum‐maximum	46–94
**Sex, *n* (%)**	
Male	144 (55.6)
**Race/ethnicity, *n* (%)**	
White	226 (87.3)
Black	16 (6.2)
Asian	17 (6.6)
Other	1 (0.4)
**Insurance type at index date, *n* (%)**	
Public insurance (NHS) only	251 (96.9)
**History of cancer other than CLL, *n* (%)**	
None	248 (95.8)
Basal cell or non‐metastatic squamous cell carcinoma of the skin	9 (3.5)
Carcinoma *in situ* of the cervix or breast	2 (0.8)
**ECOG performance status at index, *n* (%)**	
0 – Asymptomatic	58 (22.4)
1 – Symptomatic, completely ambulatory	170 (65.6)
2 – Symptomatic, <50% of waking hours spent in bed	31 (12.0)
**Rai stage at index date, *n* (%)**	
Stage 0	2 (0.8)
Stage I	7 (2.7)
Stage II	17 (6.6)
Stage III	71 (27.4)
Stage IV	83 (32.1)
Rai stage not recorded/unknown	79 (30.5)
**Binet stage at index date, *n* (%)**	
Stage A	6 (2.3)
Stage B	90 (34.8)
Stage C	144 (55.6)
Binet stage not recorded/unknown	19 (7.3)
**Comorbidities and risk factors, *n* (%)**	
Hypertension	112 (43.2)
Hyperlipidaemia	48 (18.5)
Chronic obstructive pulmonary disease	36 (13.9)
Diabetes without end‐organ damage	36 (13.9)
History of smoking/tobacco use	33 (12.7)
Depression	25 (9.7)
History of atrial fibrillation/flutter	25 (9.7)
**High‐risk prognostic factors** ^a^ **, *n* (%)**	
17p deletion	57 (22.0)
*TP53* mutations/aberrations	56 (21.6)
11q deletion	35 (13.5)
None of the above	119 (46.0)

^a^ High‐risk prognostic factors were not mutually exclusive.

Abbreviations: CLL, chronic lymphocytic leukaemia; ECOG, Eastern Cooperative Oncology Group; NHS, UK National Health Service; SD, standard deviation.

Most patients in the ibrutinib cohort had no history of cancer before CLL diagnosis (95.8%), and the majority had Rai stage III/IV (59.5%) or Binet stage C (55.6%) disease. ECOG performance status at index date showed that 22.4% of patients were asymptomatic (ECOG score 0). Genetic analyses for high‐risk prognostic factors revealed 17p deletion in 22.0% of patients, *TP53* mutations or aberrations in 21.6% and 11q deletion in 13.5%, with 46.0% having none of these variations (Table 2).

Data were also contributed for 30 patients with CLL who initiated treatment with venetoclax and 29 patients with CLL who initiated treatment with idelalisib during the case selection window (Table S1). The median age at initiation of venetoclax was 69 years, and the median age at initiation of idelalisib was 70 years. ECOG performance status at index date was predominantly 0 or 1 (90.0% and 93.1% in the venetoclax and idelalisib cohorts, respectively), and the majority had Rai stage III/IV (60.0% and 69.0% in the venetoclax and idelalisib cohorts, respectively) or Binet stage C (60.0% and 72.4% in the venetoclax and idelalisib cohorts, respectively) disease. Venetoclax was prescribed first line in one patient (3.3%), second line in 11 patients (36.7%) and third line or later in 18 patients (60.0%). Idelalisib was prescribed first line in six patients (20.7%), second line in 15 patients (51.7%) and third line or later in eight patients (27.6%).

### 3.2 Follow‐up

Median follow‐up duration in the ibrutinib cohort was 16.7 months (range, 2.8–29.2 months) from ibrutinib initiation and 62.0 months (range, 11.6–264.1 months) from CLL diagnosis. At the end of follow‐up, 95.0% of patients in this cohort were alive, and seven of the 13 deaths were CLL‐related.

### 3.3 Treatment with ibrutinib

**FIGURE 1:**
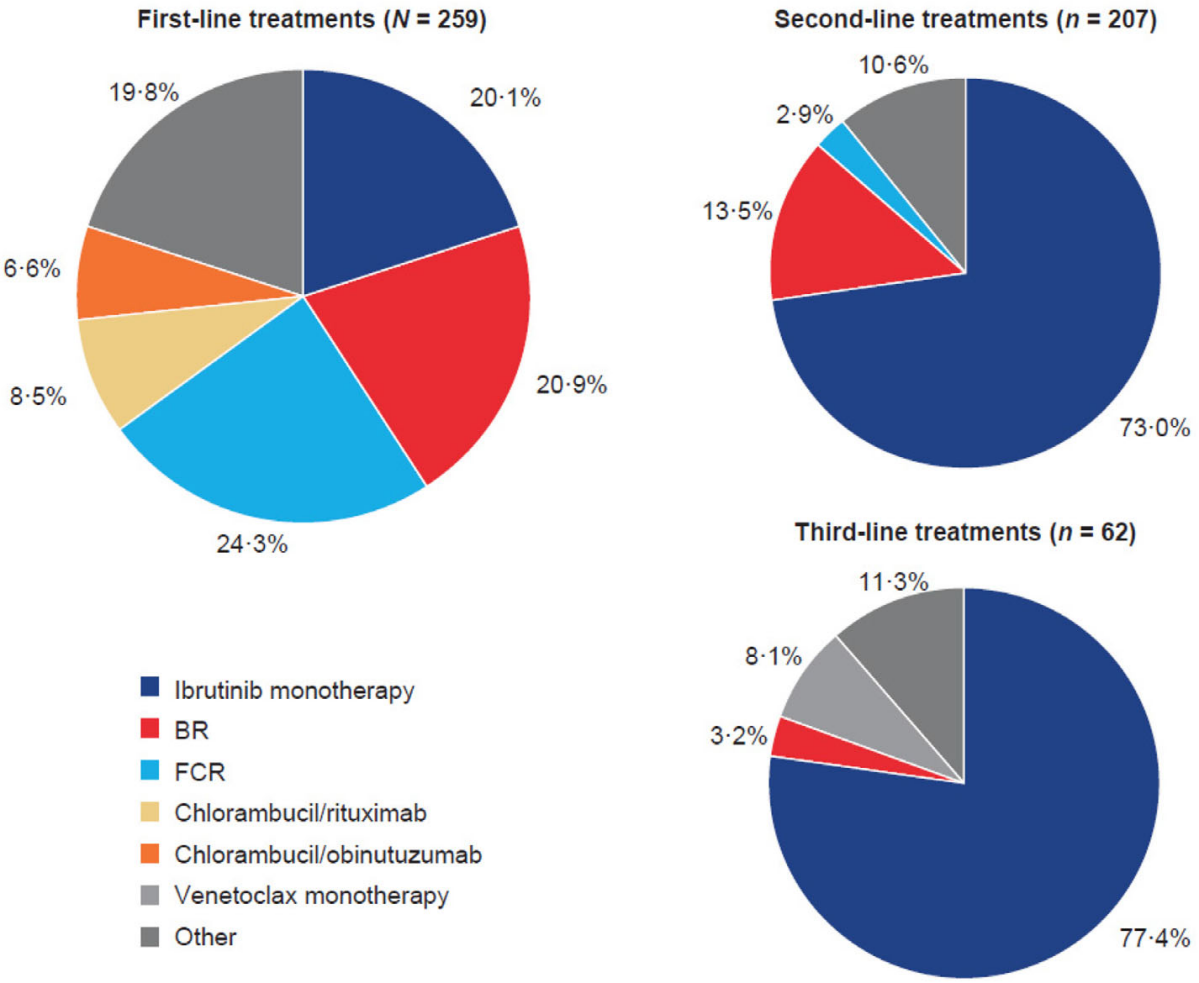
CLL treatments in patients in the ibrutinib cohort separated by line of use ‐ NB: All patients in the third‐ and second‐line therapy cohorts overlap and are included in the chart showing the previous line(s) of therapy. Abbreviations: BR, bendamustine and rituximab; CLL, chronic lymphocytic leukaemia; FCR, fludarabine, cyclophosphamide and rituximab.

Of the 259 patients in the ibrutinib cohort, the most common first‐line treatment was FCR (24.3%), followed by BR (20.9%) and ibrutinib monotherapy (20.1%). In the 207 patients in this cohort who received a second‐line treatment and the 62 patients who received a third‐line treatment, ibrutinib monotherapy was the most common choice (Figure 1).

The median time to initiation of ibrutinib treatment from CLL diagnosis in this cohort was 43.7 months (range, 0.0–249.5 months). Overall, 55 patients (21.2%) in the ibrutinib cohort received ibrutinib (either as monotherapy or in combination with another agent) in the first‐line setting; the remaining 204 (78.8%) received ibrutinib as second line or later. At the end of follow‐up, most patients were still taking ibrutinib (219; 84.6%), with a median treatment duration of 16.8 months (range, 9.1–28.3 months) (Table S2).

Among the 40 patients (15.4%) in the ibrutinib cohort who discontinued ibrutinib, the median time to discontinuation was 10.1 months (range, 0.5–28.6 months), and the most common reason for discontinuation was progressive disease (42.5% of patients, Table S3). Of those who discontinued ibrutinib, 13 patients went on to receive a further line of therapy (venetoclax in seven patients). Median time from ibrutinib discontinuation to initiation of another therapy was 0.5 months (range, 0.0–11.0 months).

Dose reduction was observed in 37 patients (14.3%) in the ibrutinib cohort, and the most cited reason was toxicity (81.1% of cases). The median time to dose reduction was 4.2 months (range, 0.8–22.2 months) and was similar across first‐line to third‐line use. Ibrutinib was temporarily discontinued in 10.4% of patients in this cohort, mostly owing to toxicity (51.9% of cases, Table S4).

### 3.4 Response to treatment

Based on patients’ best response to ibrutinib, overall response rate (complete response and partial response) was 88.4% and was similar across first‐line to fourth‐line use in the ibrutinib cohort. The clinical benefit rate (overall response and stable disease) was 91.1% and was also similar across first‐line to fourth‐line use. During treatment with ibrutinib, 23 patients (8.9%) experienced disease progression, and the median time to progression was 11.2 months (range, 2.0–28.6 months) (Table 3).

**TABLE 3:** Clinical response and disease progression during ibrutinib therapy

		**By line of therapy in which ibrutinib was initiated**			
	**Overall** **^a^** **(*N* = 259)**	**First line (*n* = 55)**	**Second line (*n* = 152)**	**Third line (*n* = 48)**	**Fourth line (*n* = 4)**
**Overall response rate** **^b^** **, *n* (%)**	**229 (88**.**4)**	**48 (87**.**3)**	**136 (89**.**5)**	**41 (85**.**4)**	**4 (100**.**0)**
**Clinical benefit rate** **^c^** **, *n* (%)**	**236 (91**.**1)**	**49 (89**.**1)**	**142 (93**.**4)**	**47 (97**.**9)**	**4 (100**.**0)**
**Patient's best response to ibrutinib based on clinical evaluation, *n* (%)**					
Complete response**^d^**	97 (37.5)	25 (45.5)	49 (32.2)	22 (45.8)	1 (25.0)
Partial response	132 (51.0)	23 (41.8)	87 (57.2)	19 (39.6)	3 (75.0)
Stable disease	7 (2.7)	1 (1.8)	6 (4.0)	6 (12.5)	0 (0.0)
Progression	15 (5.8)	2 (3.6)	7 (4.6)	0 (0.0)	0 (0.0)
Unevaluable	3 (1.2)	2 (3.6)	1 (0.7)	0 (0.0)	0 (0.0)
Unknown	5 (1.9)	2 (3.6)	2 (1.3)	1 (2.1)	0 (0.0)
**Time to best response from ibrutinib therapy initiation, months**					
Mean (SD)	11.5 (6.3)	13.3 (7.1)	10.5 (5.7)	12.9 (6.9)	12.4 (2.0)
Median	10.1	13.4	9.2	11.3	12.4
Minimum‐maximum	0.5–28.2	3.4–27.5	0.5–25.2	2.1–28.2	11–13.8
Missing/other/unknown (*n*, %)	102 (39.4)	23 (41.8)	56 (36.8)	21 (43.8)	2 (50.0)
**Total patients progressed (any time after ibrutinib initiation), *n* (%)**	**23 (8**.**9)**	**3 (5**.**5)**	**14 (9**.**2)**	**5 (10**.**4)**	**1 (25**.**0)**
**Time to disease progression from ibrutinib initiation, months**					
Mean (SD)	12.4 (7.9)	14.3 (6.5)	9.6 (6.6)	17.4 (10.0)	20.9
Median	11.2	13.1	7.8	19.7	20.9
Minimum‐maximum	2.0–28.6	8.5–21.3	2.0–28.6	2.9–26.3	20.9–20.9

^a^ Overall measure was assessed based on initiation of ibrutinib therapy at any time in the follow‐up period, regardless of the therapy line in which it was initiated.

^b^ Overall response rate: complete response + partial response.

^c^ Clinical benefit rate: complete response + partial response + stable disease.

^d^ Based on information contained within medical records as reviewed by participating clinicians or designated clinical staff. Abbreviation: SD, standard deviation.

In the venetoclax cohort, the overall rate of response to venetoclax based on patients’ best response was 90.0%, and the clinical benefit rate was 96.7%. A complete response was recorded in 56.7% of patients, with a partial response in 33.3% of patients. No patients in this cohort experienced disease progression while taking venetoclax during the study period.

For the idelalisib cohort, the overall rate of response based on patients’ best response was 93.1%, and the clinical benefit rate was 100%. A complete response was recorded in 31.0% of patients, with a partial response in 62.1% of patients. Four patients (13.8%) in this cohort experienced disease progression while taking idelalisib during the study period.

### 3.5 AEs

**FIGURE 2:**
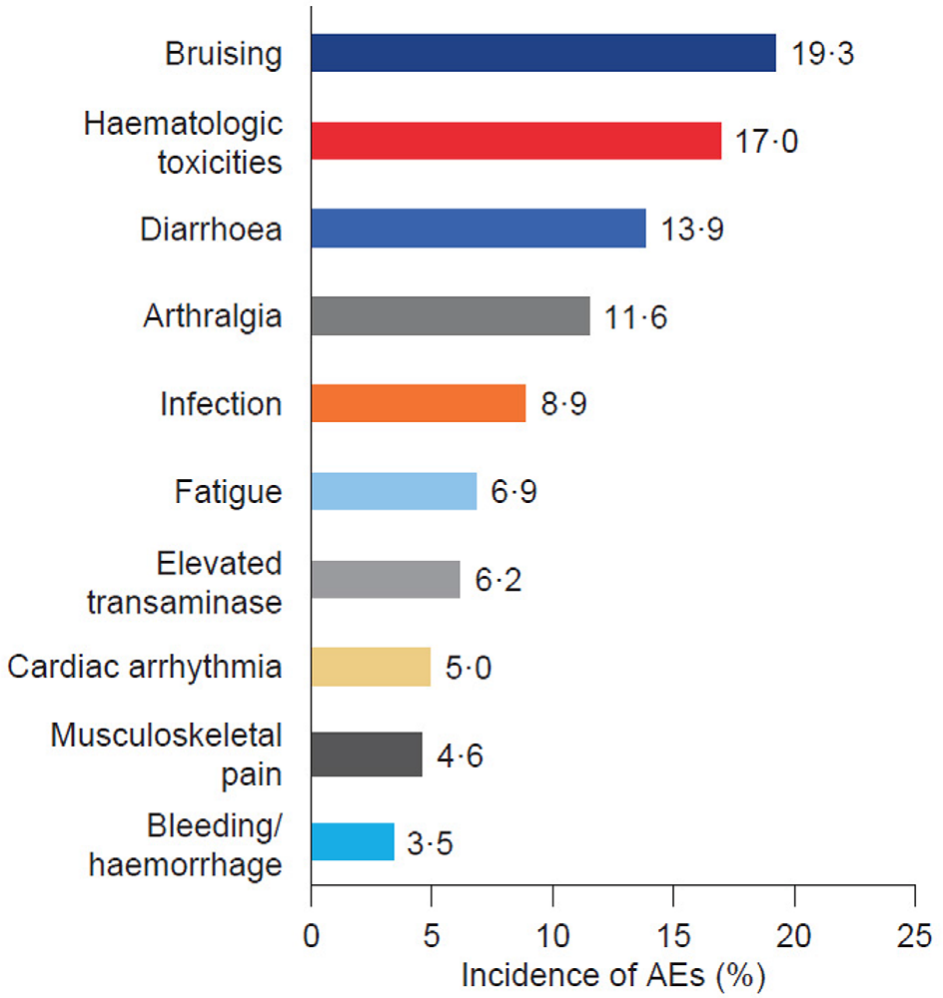
Incidence of most common AEs during ibrutinib therapy. Haematologic toxicities included cytopenias such as anaemia, lymphopenia, lymphocytosis, neutropenia and thrombocytopenia Abbreviation: AE, adverse event.

In the ibrutinib cohort, AEs were recorded in 151 of 259 patients (58.3%) during ibrutinib treatment. The most common AEs were bruising (19.3% of patients), cytopenias (17.0% of patients), diarrhoea (13.9% of patients) and arthralgia (11.6% of patients) (Figure 2). Infections were recorded for 23 patients (8.9%) during ibrutinib therapy, and 13 patients were hospitalised as a result. Of the nine patients (3.5%) who experienced bleeding, the event was considered major (e.g. gastrointestinal bleeding, haematuria) in five patients. During ibrutinib therapy, 12 patients (4.6%) experienced atrial fibrillation (AF), of whom two had a history of AF. Toxicity was cited as the reason for discontinuation in nine patients (3.5%).

In the venetoclax cohort, AEs were recorded in 16 of 30 patients (53.3%) during venetoclax treatment. The most common AEs in this cohort were cytopenias (30.0% of patients), infection (23.3% of patients) and fatigue (16.7% of patients) (Table S5).

In the idelalisib cohort, AEs were recorded in 22 of 29 patients (75.9%) during idelalisib treatment. The most common AEs associated with idelalisib treatment were infection (27.6% of patients), cytopenias (24.1% of patients) and colitis (20.7% of patients) (Table S5).

### 3.6 Survival

**FIGURE 3:**
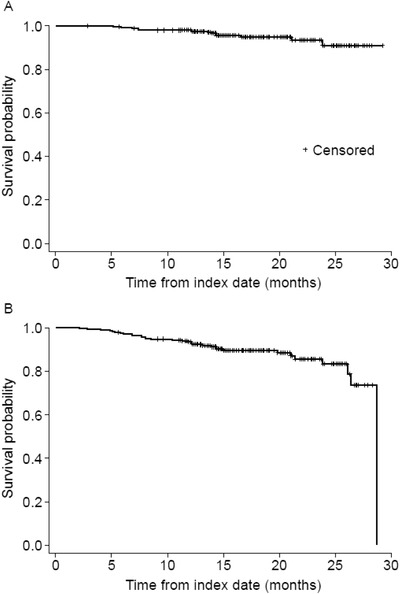
Kaplan‐Meier estimates of (A) overall survival and (B) progression‐free survival from the start of ibrutinib therapy (any line)

In the ibrutinib cohort, the 12‐month and 24‐month OS rates from the start of ibrutinib treatment were 98.0% (95% confidence interval [CI]: 96.3–99.7) and 92.5% (95% CI: 88.5–96.6), respectively (Figure 3). The 12‐month and 24‐month PFS rates from the start of ibrutinib treatment were 93.3% (95% CI: 90.2–96.4) and 83.7% (95% CI: 77.4–90.0), respectively (Figure 3, Table S6). There were insufficient data to perform survival analyses on the venetoclax and idelalisib cohorts.

### 3.7 Healthcare resource use

**FIGURE 4:**
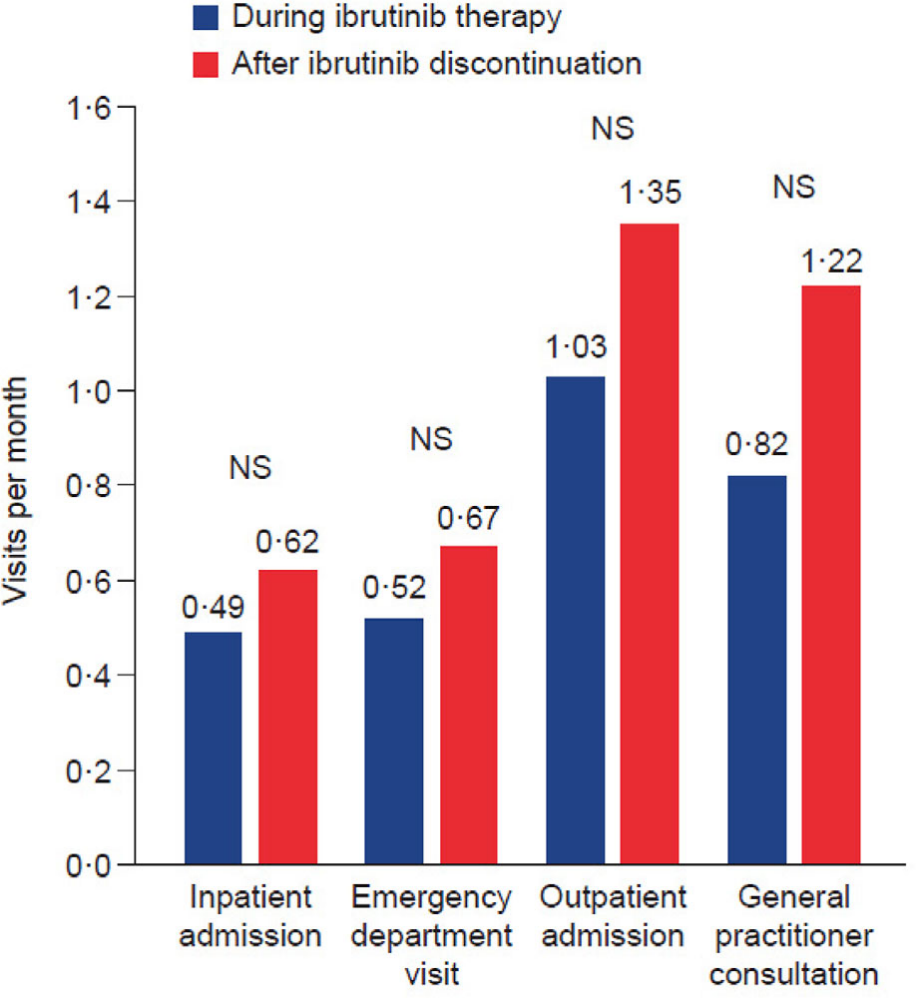
Average number of visits per patient per month (among patients with more than one visit), during ibrutinib therapy and after discontinuation (*n* = 37) Abbreviation: NS, not significant.

In the 37 patients in the ibrutinib cohort who discontinued ibrutinib and had valid healthcare resource utilization data, the median duration of follow‐up was 8.8 months (range, 1.0–29.0 months) during ibrutinib therapy and 1.9 months (range, 0.0–24.0 months) after discontinuation. Healthcare resource utilization after ibrutinib discontinuation did not differ significantly from utilization during ibrutinib therapy in these patients. Standardised monthly rates of inpatient admission, emergency department visits, outpatient visits and general practitioner consultations were, however, numerically higher after ibrutinib discontinuation (Figure 4, Table S7). There were insufficient data to examine pre‐ and post‐discontinuation healthcare resource utilization in the venetoclax and idelalisib cohorts.

## 4 DISCUSSION

This retrospective study provides insights into the real‐world treatment patterns and outcomes of patients with CLL in the UK treated with oral targeted therapies. Ibrutinib was most often prescribed as second‐line or third‐line treatment and was the third most frequently prescribed first‐line treatment during the study period. The chemoimmunotherapies FCR and BR were the first and second most common first‐line treatments, respectively. These patterns may reflect the NICE guidelines, which recommend first‐line ibrutinib only for patients with 17p deletion or *TP53* mutation in whom chemoimmunotherapy is unsuitable [11].

Estimates of 12‐month OS after initiation of ibrutinib in relapsed/refractory CLL were 83.8% in the 2016 UK CLL Forum observational study [14], 100% in a UK single‐centre observational study [19] and 90.0% in the RESONATE trial [8], compared with 98.0% across all lines of treatment in the present study. Estimates of 24‐month OS and PFS were 98% and 89%, respectively, in the frontline RESONATE‐2 trial [9], compared with 92.5% and 83.7% across all lines of treatment in the present study. While the estimates of OS and PFS after ibrutinib initiation were similar across first‐line to third‐line use in the present study, the modest median duration of ibrutinib therapy (15.9 months) must be considered while interpreting these results. Future long‐term studies will provide additional insight on the effect of ibrutinib on survival outcomes in real‐world clinical practice.

More than half of the patients prescribed ibrutinib experienced AEs during treatment (58.3%). This is consistent with the 2016 UK CLL Forum report, which found that 56.5% of patients experienced an ibrutinib‐related AE [14]. The high incidence of bruising, haematological toxicities, diarrhoea and arthralgia seen in the present study is consistent with results from the RESONATE and RESONATE‐2 trials, as well as previous real‐world observational studies [8, 9, 18, 19].

Infection is a common AE associated with ibrutinib therapy [23, 24]. In the present ibrutinib cohort, 23 patients had records of infection while taking ibrutinib, and 13 of these were hospitalised as a result. Guidance from the UK Medicines and Healthcare products Regulatory Agency recommends antimicrobial prophylaxis for patients at an increased risk of opportunistic infections while taking ibrutinib [25]. Physicians’ adherence to this guideline may explain why infections were observed in only 3.5% of patients in the present study, a much lower proportion than in the RESONATE trial (70.0% of patients in the ibrutinib group) [8].

In the ibrutinib cohort, 3.5% of patients experienced bleeding, and 4.6% of patients experienced AF, consistent with the known side effect profile of ibrutinib [18, 19]. The incidence of major bleeding events highlights the need for extra care when prescribing ibrutinib to patients with a history of bleeding or those taking anticoagulants. AF can have serious clinical consequences, and co‐managing AF and CLL treatment with ibrutinib can be a complex matter owing to the use of anticoagulant therapy for AF alongside ibrutinib, which is known to predispose to bleeding. This can lead to discontinuation of ibrutinib therapy [26], as was the case for four patients in the present study. The occurrence of AF in several patients without a history of the condition highlights the need for careful consideration of predisposing factors such as ischaemia and hypertension when prescribing ibrutinib.

A smaller proportion of patients permanently discontinued ibrutinib therapy in the present study than in previous studies; the majority of ibrutinib discontinuations were due to disease progression [12, 18, 27, 28]. For example, the rate of discontinuation due to AEs in the present study was lower than in the final analysis of the RESONATE clinical trial (3.5% compared with 16.4%, respectively). In the present study, CLL progression was the reason for discontinuation in 42.5% of patients, a greater proportion than seen in observational studies in the USA [18] and the 2016 UK CLL Forum report [14]. These differences may be due to physicians opting to mitigate AEs with dose reductions or temporary discontinuation. Poor ibrutinib dose adherence has been reported to reduce PFS in CLL [27], suggesting that toxicity may limit efficacy.

The data presented here should be interpreted with caution, owing to the relatively short median duration of follow‐up from treatment initiation. OS and PFS data were not mature because of the recent observation window and the slow progression of CLL. Additionally, the median follow‐up duration after discontinuation of ibrutinib was short (1.9 months), which limited the usefulness of comparisons between pre‐ and post‐discontinuation healthcare utilization. The quality and completeness of data entered into the data collection form were limited to information available in the medical records held by participating physicians. The data were entered directly by treating physicians or qualified staff delegates and therefore may be subject to data entry errors, although all data were checked for internal consistency. The numbers of patients in the venetoclax and idelalisib cohorts was small because these patients were not the primary focus of this study, and therefore caution should be used when interpreting these results. Future chart reviews that focus on patients treated with venetoclax and idelalisib can help to re‐evaluate this patient population and provide further insight into their use in clinical practice.

Results from this study provide information on the real‐world safety and tolerability of ibrutinib and demonstrate that use of ibrutinib quickly became commonplace in routine UK clinical practice following its approval in January 2017, particularly in second‐line or later treatment of CLL. Despite evidence that oral ibrutinib offers significant survival benefits compared with intravenous chemoimmunotherapy, AEs remain a common occurrence. Physicians often choose to mitigate these AEs through dose reduction or temporary discontinuation, although permanent discontinuation of ibrutinib therapy due to AEs is also observed. Because outcomes of patients who discontinue ibrutinib therapy are generally poor [12], next‐generation BTK inhibitors with more favourable tolerability profiles could provide an improved treatment option for patients with CLL who are unable to tolerate ibrutinib. In such patients, it would be preferable to switch to a tolerable BTK inhibitor as opposed to changing the class of treatment prematurely and losing a valuable line of therapy.

## AUTHOR CONTRIBUTIONS

Peter Hillmen, Jing Xie, Alan S. M. Yong, Catherine Waweru, Thuy Anh Sorof, Ravi K. Goyal and Keith L. Davis contributed to the design of the research study. Jing Xie, RKG, KLD and CW developed and validated the survey instrument. Jing Xie, Alan S. M. Yong, Thuy Anh Sorof, Ravi K. Goyal and Keith L. Davis reviewed and analysed the data. The content of the manuscript was agreed upon by all authors, and all authors contributed to manuscript development. All authors approved the final version of the manuscript before submission. An AstraZeneca team reviewed this publication, without influencing the opinion of the authors, to ensure medical and scientific accuracy and to protect intellectual property. The corresponding author had access to all study protocols and results and had the final responsibility for the decision to submit the manuscript for publication.

## Supporting information



Supporting Information
