# Supplementary material for: Real‐world treatment patterns, adverse events and clinical outcomes in patients with chronic lymphocytic leukaemia treated with ibrutinib in the UK
Source: EJHaem. 2021 Mar 13;2(2):219–27. doi: 10.1002/jha2.174 (PMC9175847; doi:10.1002/jha2.174)
Supplement: Supplementary file 1 — Supporting Information [file JHA2-2-219-s001.docx]

## Table S1. Baseline demographics and clinical characteristics of patients with CLL in the venetoclax and idelalisib cohorts.

| **Measures** | **Venetoclax  (*N* = 30)** | **Idelalisib**  **(*N* = 29)** |
| --- | --- | --- |
| **Age at index date, years** |  |  |
| Mean (SD) | 67·8 (7·9) | 70·2 (8·3) |
| Median | 69 | 70 |
| Min–max | 49–85 | 53–86 |
| **Sex, *n* (%)** |  |  |
| Male | 16 (53·3) | 16 (55·2) |
| **Race/ethnicity, *n* (%)** |  |  |
| White | 26 (86·7) | 27 (93·1) |
| Black | 1 (3·3) | 0 (0·0) |
| Asian | 3 (10·0) | 2 (6·9) |
| Other | 0 (0·0) | 0 (0·0) |
| **Insurance type at index date, *n* (%)** |  |  |
| Public insurance (NHS) only | 29 (96·7) | 28 (96·6) |
| **History of cancer other than CLL, *n* (%)** |  |  |
| None | 30 (100·0) | 28 (96·6) |
| Basal cell or non-metastatic squamous cell carcinoma of the skin | 0 (0·0) | 1 (3·5) |
| Carcinoma *in situ* of the cervix or breast | 0 (0·0) | 0 (0·0) |
| **ECOG performance status at index, *n* (%)** |  |  |
| 0 – Asymptomatic | 7 (23·3) | 7 (24·1) |
| 1 – Symptomatic, completely ambulatory | 20 (66·7) | 20 (69·0) |
| 2 – Symptomatic, < 50% of waking hours spent in bed | 30 (10·0) | 2 (6·9) |
| **Rai stage at index date, *n* (%)** |  |  |
| Stage 0 | 0 (0·0) | 0 (0·0) |
| Stage I | 0 (0·0) | 1 (3·5) |
| Stage II | 3 (10·0) | 0 (0·0) |
| Stage III | 5 (16·7) | 7 (24·1) |
| Stage IV | 13 (43·3) | 13 (44·8) |
| Rai stage not recorded/unknown | 9 (30·0) | 8 (27·6) |
| **Binet stage at index date, *n* (%)** |  |  |
| Stage A | 0 (0·0) | 1 (3·5) |
| Stage B | 10 (33·3) | 7 (24·1) |
| Stage C | 28 (60·0) | 21 (72·4) |
| Binet stage not recorded/unknown | 2 (6·7) | 0 (0·0) |
| **Comorbidities and risk factors, *n* (%)** |  |  |
| Hypertension | 13 (43·3) | 14 (48·3) |
| Hyperlipidaemia | 7 (23·3) | 7 (24·1) |
| Chronic obstructive pulmonary disease | 3 (10·0) | 4 (13·8) |
| Diabetes without end-organ damage | 3 (10·0) | 2 (6·9) |
| History of smoking/tobacco use | 3 (10·0) | 3 (10·3) |
| Depression | 4 (13·3) | 2 (6·9) |
| History of atrial fibrillation/flutter | 6 (20·0) | 10 (34·5) |
| **High-risk prognostic factors^a^, *n* (%)** |  |  |
| 17p deletion | 8 (26·7) | 15 (51·7) |
| *TP53* mutations/aberrations | 10 (33·3) | 8 (27·6) |
| 11q deletion | 6 (20·0) | 2 (6·9) |
| None of the above | 7 (23·3) | 6 (20·7) |
| **Line of therapy, *n* (%)** |  |  |
| First line (from CLL diagnosis) | 1 (3·3) | 6 (20·7) |
| Second line | 11 (36·7) | 15 (51·7) |
| Third line | 13 (43·3) | 7 (24·1) |
| Fourth line | 5 (16·7) | 0 (0·0) |
| Fifth line | 0 (0·0) | 1 (3·5) |

^a^High-risk prognostic factors were not mutually exclusive. CLL, chronic lymphocytic leukaemia; ECOG, Eastern Cooperative Oncology Group; NHS, UK National Health Service; SD, standard deviation.

### Table S2. Treatment characteristics of the ibrutinib cohort by line of therapy.

|  |  | **By line of therapy in which ibrutinib was initiated** | | | |
| --- | --- | --- | --- | --- | --- |
|  | **Overall^a^**  **(*N* = 259)** | **First line**  **(*n* = 55)** | **Second line**  **(*n* = 152)** | **Third line**  **(*n* = 48)** | **Fourth line**  **(*n* = 4)** |
| **Total patients initiating ibrutinib, %** | **100**·**0** | **21**·**2** | **58**·**7** | **18**·**5** | **1**·**5** |
| **Time to initiation of ibrutinib^b^, months** |  |  |  |  |  |
| Mean (SD) | 55·1 (48·5) | 15·0 (28·4) | 28·6 (26·6) | 22·6 (20·6) | 39·6 (39·6) |
| Median | 43·7 | 3·0 | 22·7 | 19·7 | 31·5 |
| Interquartile range | 17·8–80·8 | 1·1–13·6 | 12·0–39·6 | 7·4–31·6 | 7·6–71·6 |
| Min–max | 0·0–249·5 | 0·0–123·7 | 0·0–242·2 | 0·2–118·7 | 6·4–88·9 |
| **Ibrutinib therapy status at end of follow-up, *n* (%)** |  |  |  |  |  |
| Discontinued | 40 (15·4) | 8 (14·5) | 22 (14·5) | 9 (18·8) | 1 (25·0) |
| Ongoing | 219 (84·6) | 47 (85·5) | 130 (85·5) | 39 (81·3) | 3 (75·0) |
| **Duration of ibrutinib therapy, months** |  |  |  |  |  |
| *Among patients who discontinued therapy* |  |  |  |  |  |
| Mean (SD) | 11·0 (7·5) | 11·1 (7·9) | 9·1 (6·2) | 14·1 (8·8) | 23·0 |
| Median | 10·1 | 9·3 | 8·6 | 13·6 | 23·0 |
| Interquartile range | 5·0–13·8 | 6·1–17·3 | 4·8–12·0 | 5·8–19·8 | 23·0–23·0 |
| Min–max | 0·5–28·6 | 0·5–23·3 | 0·5–28·6 | 2·8–26·7 | 23·0֪–23·0 |
| *Among patients whose therapy was ongoing at end of follow-up* |  |  |  |  |  |
| Mean (SD) | 17·8 (4·7) | 18·4 (4·4) | 17·3 (4·4) | 18·7 (6·0) | 16·2 (2·2) |
| Median | 16·8 | 17·5 | 16·2 | 15·9 | 16·8 |
| Interquartile range | 14·0–21·1 | 14·8–22·8 | 13·8–20·9 | 13·6–25·6 | 13·8–18·1 |
| Min–max | 9·1–28·3 | 11·8–27·8 | 9·1–27·0 | 11·3–28·3 | 13·8–18·1 |
| *Among all patients* |  |  |  |  |  |
| Mean (SD) | 16·7 (5·8) | 17·4 (5·6) | 16·1 (5·5) | 17·8 (6·7) | 17·9 (3·8) |
| Median | 15·9 | 17·2 | 15·9 | 15·9 | 17·5 |
| Interquartile range | 13·1–20·9 | 14·0–22·1 | 12·8–20·0 | 13·1–25·6 | 15·3–20·6 |
| Min–max | 0·5–28·6 | 0·5–27·8 | 0·5–28·6 | 2·8–28·3 | 13·8–23·0 |

^a^Overall measure was assessed based on initiation of ibrutinib therapy at any time in the follow-up period, regardless of the therapy line in which it was initiated; ^b^Time to initiation for second and later lines were assessed from the end of the previous line of therapy.
CLL, chronic lymphocytic leukaemia; SD, standard deviation.

### Table S3. Time to and reason for ibrutinib discontinuation in the ibrutinib cohort.

|  |  | | **By line of therapy in which ibrutinib was initiated** | | | |
| --- | --- | --- | --- | --- | --- | --- |
|  | **Overall^a^**  **(*N* = 259)** | **First line**  **(*n* = 55)** | | **Second line**  **(*n* = 152)** | **Third line**  **(*n* = 48)** | **Fourth line**  **(*n* = 4)** |
| **Patients who discontinued ibrutinib, *n* (%)** | **40 (15**·**4)** | **8 (15**·**1)** | | **22 (14**·**5)** | **9 (18**·**8)** | **1 (25**·**0)** |
| **Time to discontinuation, months** |  |  | |  |  |  |
| Mean (SD) | 11 (7·5) | 11 (7·9) | | 9·1 (6·2) | 14·1 (8·8) | 23·0 (-) |
| Median (crude estimate) | 10·1 | 9·3 | | 8·6 | 13·6 | 23·0 |
| Interquartile range | 5·0–13·8 | 6·1–17·3 | | 4·8–12·0 | 5·8–19·8 | 23·0–23·0 |
| Min–max | 0·5–28·6 | 0·5–23·3 | | 0·5–28·6 | 2·8–26·7 | 23·0–23·0 |
| **Reason(s) for discontinuation^b^, *n* (%)** |  |  | |  |  |  |
| Toxicity | 9 (22·5) | 2 (25·0) | | 5 (22·7) | 2 (22·2) | 0 (0·0) |
| Haematologic toxicity | 3 (7·5) | 1 (12·5) | | 1 (4·6) | 1 (11·1) | 0 (0·0) |
| Infection | 3 (7·5) | 1 (12·5) | | 0 (0·0) | 2 (22·2) | 0 (0·0) |
| Atrial fibrillation | 4 (10·0) | 1 (12·5) | | 3 (13·6) | 0 (0·0) | 0 (0·0) |
| Congestive heart failure | 1 (2·5) | 1 (12·5) | | 0 (0·0) | 0 (0·0) | 0 (0·0) |
| Pneumonitis | 1 (2·5) | 0 (0·0) | | 0 (0·0) | 1 (11·1) | 0 (0·0) |
| Bleeding/haemorrhage | 2 (5·0) | 1 (12·5) | | 0 (0·0) | 1 (11·1) | 0 (0·0) |
| Arthralgia/myalgia | 1 (2·5) | 0 (0·0) | | 0 (0·0) | 1 (11·1) | 0 (0·0) |
| Dermatological toxicity | 1 (2·5) | 0 (0·0) | | 1 (4·6) | 0 (0·0) | 0 (0·0) |
| Progressive disease | 17 (42·5) | 3 (37·5) | | 8 (36·4) | 5 (55·6) | 1 (10·0) |
| Richter transformation | 1 (2·5) | 0 (0·0) | | 1 (4·6) | 0 (0·0) | 0 (0·0) |
| Secondary malignancy or second primary malignancy | 3 (7·5) | 0 (0·0) | | 2 (9·1) | 1 (11·1) | 0 (0·0) |
| Physician’s or patient’s preference | 6 (15·0) | 1 (12·5) | | 4 (18·2) | 1 (11·1) | 0 (0·0) |
| Death | 3 (7·5) | 2 (25·0) | | 2 (9·1) | 2 (22·2) | 0 (0·0) |
| Other | 3 (7·5) | 1 (12·5) | | 2 (9·1) | 0 (0·0) | 0 (0·0) |
| Abdominal surgery followed by MI | 1 (2·5) | 0 (0·0) | | 1 (4·6) | 0 (0·0) | 0 (0·0) |
| Extreme breathlessness | 1 (2·5) | 1 (12·5) | | 0 (0·0) | 0 (0·0) | 0 (0·0) |
| Went on to stem cell transplant | 1 (2·5) | 0 (0·0) | | 1 (4·6) | 0 (0·0) | 0 (0·0) |

^a^Overall measure was assessed based on initiation of ibrutinib therapy at any time in the follow–up period, regardless of the therapy line in which it was initiated. ^b^Reasons for discontinuation were not mutually exclusive.
MI, myocardial infarction; SD, standard deviation.

### Table S4. Time to and reasons for ibrutinib dose reduction and temporary discontinuation in the ibrutinib cohort.

|  |  | **By line of therapy of ibrutinib initiation** | | |
| --- | --- | --- | --- | --- |
|  | **Overall^a^**  **(*N* = 259)** | **First line**  **(*n* = 55)** | **Second line**  **(*n* = 152)** | **Third line**  **(*n* = 48)** |
| **Patients who experienced dose reduction, *n* (%)** | **37 (14**·**3)** | **8 (15**·**1)** | **21 (13**·**8)** | **8 (16**·**7)** |
| **Time to dose reduction, months** |  |  |  |  |
| Mean (SD) | 6·7 (6·2) | 5·6 (5·7) | 7·2 (6·3) | 6·4 (7·0) |
| Median | 4·2 | 3·4 | 5·5 | 2·9 |
| Interquartile range | 2·3–9·0 | 2·6–6·8 | 2·8–9·0 | 0·8–20·0 |
| Min–max | 0·8–22·2 | 1·1–18·1 | 0·9–22·2 | 0·8–20·0 |
| **Reason(s) for dose reduction, *n* (%)** |  |  |  |  |
| Toxicity | 30 (81·1) | 8 (100·0) | 16 (76·2) | 6 (75·0) |
| Haematologic toxicity | 21 (56·8) | 4 (50·0) | 12 (57·1) | 5 (62·5) |
| Atrial fibrillation | 3 (8·1) | 2 (25·0) | 1 (4·8) | 0 (0·0) |
| Secondary or second primary malignancy | 1 (2·7) | 0 (0·0) | 1 (4·8) | 0 (0·0) |
| Physician’s or patient’s preference | 8 (21·6) | 0 (0·0) | 6 (26·8) | 2 (25) |
| **Patients who discontinued ibrutinib temporarily,^b^ *n* (%)** | **27 (10**·**4)** | **8 (14**·**5)** | **14 (9**·**2)** | **5 (10**·**4)** |
| **Time to temporary discontinuation,^b^ months** |  |  |  |  |
| Mean (SD) | 8·0 (5·6) | 9·4 (5·9) | 6·6 (3·7) | 9·5 (9·4) |
| Median | 6·4 | 9·7 | 5·6 | 6·4 |
| Interquartile range | 3·8–11·2 | 3·65–13·1 | 4·0–8·9 | 3·0–13·0 |
| Min–max | 0·8–24·3 | 3·0–19·5 | 1·6–13·8 | 0·8–24·2 |
| **Reason(s) for temporary discontinuation,^b^ *n* (%)** |  |  |  |  |
| Hold for invasive procedure | 9 (33·3) | 3 (37·5) | 3 (21·4) | 3 (60·0) |
| Toxicity | 14 (51·9) | 4 (50·0) | 8 (57·1) | 2 (40·0) |
| Haematologic toxicity | 5 (18·5) | 2 (25·0) | 3 (21·4) | 0 (0·0) |
| Atrial fibrillation | 5 (18·5) | 2 (25·0) | 3 (21·4) | 0 (0·0) |
| Physician’s or patient’s preference | 2 (7·4) | 0 (0·0) | 2 (14·3) | 0 (0·0) |

^a^Overall measure was assessed based on initiation of ibrutinib therapy at any time in the follow-up period, regardless of the therapy line in which it was initiated. ^b^Temporary discontinuation was defined as a break of therapy with > 14 days duration.
SD, standard deviation.

## Table S5. Reported incidence of AEs incurred in patients in the venetoclax and idelalisib cohorts across all lines of therapy.

| **Patients who incurred AE, *n* (%)** | **Venetoclax  (*N* = 30)** | **Idelalisib**  **(*N* = 29)** |
| --- | --- | --- |
| Any | 16 (53·3) | 22 (75·9) |
| Arthralgia | 1 (3·3) | 3 (10·3) |
| Bleeding/haemorrhage | 0 (0·0) | 1 (3·4) |
| Minor (e.g., contusion) | 0 (0·0) | 1 (3·4) |
| Major (e.g., GI bleed, haematuria) | 0 (0·0) | 0 (0·0) |
| Bruising | 2 (6·7) | 0 (0·0) |
| Chronic pain | 1 (3·3) | 0 (0·0) |
| Colitis | 2 (6·7) | 6 (20·7) |
| Constipation | 1 (3·3) | 1 (3·4) |
| Cytopenias | 9 (30·0) | 7 (24·1) |
| Anaemia | 6 (20·0) | 4 (13·8) |
| Lymphopenia | 0 (0·0) | 0 (0·0) |
| Lymphocytosis | 0 (0·0) | 0 (0·0) |
| Neutropenia | 8 (26·7) | 6 (20·7) |
| Thrombocytopenia | 3 (10·0) | 4 (13·8) |
| Other | 0 (0·0) | 0 (0·0) |
| Dehydration | 1 (3·3) | 1 (3·4) |
| Diarrhoea | 2 (6·7) | 6 (20·7) |
| Oedema peripheral | 2 (6·7) | 0 (0·0) |
| Elevated transaminase | 1 (3·3) | 4 (13·8) |
| Fatigue | 5 (16·7) | 4 (13·8) |
| Hyperkalaemia | 1 (3·3) | 0 (0·0) |
| Hyperphosphataemia | 2 (6·7) | 0 (0·0) |
| Hyperuricaemia | 2 (6·7) | 0 (0·0) |
| Hypocalcaemia | 1 (3·3) | 0 (0·0) |
| Infection | 7 (23·3) | 8 (27·6) |
| Cytomegalovirus | 0 (0·0) | 1 (3·4) |
| Pneumonia | 3(10·0) | 4 (13·8) |
| Sinusitis | 0 (0·0) | 0 (0·0) |
| Skin infection/rash | 1 (3·3) | 1 (3·4) |
| Upper respiratory tract infection | 1 (3·3) | 3 (10·3) |
| Other infection | 3(10·0) | 1 (3·4) |
| Muscle spasms | 1 (3·3) | 0 (0·0) |
| Musculoskeletal pain | 1 (3·3) | 1 (3·4) |
| Nausea | 2 (6·7) | 0 (0·0) |
| Pyrexia | 1 (3·3) | 0 (0·0) |
| Tumour lysis syndrome | 2 (6·7) | 0 (0·0) |
| Other | 1 (3·3) | 1 (3·4) |

### Table S6. Kaplan–Meier estimates of OS and PFS in the ibrutinib cohort from start of ibrutinib therapy, by line of ibrutinib initiation.

|  |  | **By line of therapy in which ibrutinib was initiated** | | |
| --- | --- | --- | --- | --- |
| **OS, *n* (%)** | **From start of ibrutinib therapy, any line (*N* = 259)** | **From start of ibrutinib as first line (*n* = 55)** | **From start of ibrutinib as second line (*n* = 152)** | **From start of ibrutinib in third line (*n* = 48)** |
| Patients with event | 13 (5·0) | 4 (7·3) | 4 (2·6) | 5 (10·4) |
| Patients censored | 246 (95·0) | 51 (92·7) | 148 (97·4) | 43 (89·6) |
| **OS time, months (95% CI)** |  |  |  |  |
| Median | Not reached (NE, NE) | Not reached, (23·8, NE) | Not reached (NE, NE) | Not reached (NE, NE) |
| **OS, % (95% CI)** |  |  |  |  |
| 12-month rate | 98·0 (96·3–99·7) | 96·3 (91·1–100·0) | 99·3 (97·9–100·0) | 95·8 (90·1–100·0) |
| 24-month rate | 92·5 (88·5–96·6) | 84·9 (69·5–100·0) | 96·0 (92·0–99·9) | 87·2 (76·6–97·8) |
| **PFS, n (%)** | **From start of ibrutinib therapy, any line**  **(*N* = 258)^a^** | **From start of ibrutinib as first line (*n* = 55)** | **From start of ibrutinib as second line (*n* = 152)** | **From start of ibrutinib in third line (*n* = 47)^a^** |
| Patients with event | 32 (12·4) | 6 (10·9) | 17 (11·2) | 8 (17·0) |
| Patients censored | 226 (87·6) | 49 (89·1) | 135 (88·8) | 39 (83·0) |
| **PFS, months (95% CI)** |  |  |  |  |
| Median | 28·7 (NE, NE) | NE (23·8, NE) | 28·6 (NE, NE) | NE (26·3, NE) |
| **PFS rate, % (95% CI)** |  |  |  |  |
| 12-month rate | 93·3 (90·2–96·4) | 94·4 (88·2–100·0) | 92·5 (88·3–96·8) | 93·6 (86·5–100·0) |
| 24-month rate | 83·7 (77·4–90·0) | 81·1 (64·5–96·7) | 87·5 (81·6–93·4) | 82·7 (69·1–96·3) |

^a^PFS data was unavailable for one patient.

CI, confidence interval; NE, not estimable; OS, overall survival; PFS, progression-free survival.

### Table S7. Healthcare resource utilization of patients in the ibrutinib cohort during and after ibrutinib therapy, among patients who discontinued.

| **Total patients who discontinued ibrutinib** | **40** |  |  |
| --- | --- | --- | --- |
| **Total patients with valid data on resource use^a^** | **37** |  |  |
|  | **During ibrutinib therapy** | **After ibrutinib discontinuation** | ***P* value^b^** |
| **Duration of follow-up, months** |  |  |  |
| Mean (SD) | 10·6 (7·6) | 5·5 (6·3) |  |
| Median | 8·8 | 1·9 |  |
| Min–max | 1·0–29·0 | 0–24·0 |  |
| **Inpatient hospitalizations** |  |  |  |
| *Had ≥ 1 hospitalization, n (%)* |  |  |  |
| Yes | 19 (51·4) | 12 (32·4) |  |
| No | 17 (45·9) | 23 (62·2) |  |
| Unknown | 1 (2·7) | 2 (5·4) |  |
| *Number of hospitalizations per month (among patients with ≥ 1 hospitalization)* |  |  |  |
| Mean (SD) | 0·49 (0·61) | 0·62 (0·77) |  |
| Median | 0·20 | 0·24 | 0·0781 |
| Min–max | 0·04–2·00 | 0·08–2·56 |  |
| **Emergency department visits** |  |  |  |
| *Had ≥ 1 emergency department visit, n (%)* |  |  |  |
| Yes | 16 (43·2) | 6 (16·2) |  |
| No | 20 (54·1) | 31 (83·8) |  |
| Unknown | 1 (2·7) | 0 (0·0) |  |
| *Number of visits per month (among patients with ≥ 1 visit)* |  |  |  |
| Mean (SD) | 0·52 (0·64) | 0·67 (0·77) |  |
| Median | 0·20 | 0·35 | 0·0938 |
| Min–max | 0·07–2·00 | 0·08–2·04 |  |
| **Outpatient visits** |  |  |  |
| *Had ≥ 1 outpatient visit, n (%)* |  |  |  |
| Yes | 29 (78·4) | 13 (35·1) |  |
| No | 8 (21·6) | 24 (64·9) |  |
| Unknown | 0 (0·0) | 0 (0·0) |  |
| *Number of visits per month (among patients with ≥ 1 visit)* |  |  |  |
| Mean (SD) | 1·03 (0·90) | 1·35 (0·98) |  |
| Median | 0·98 | 1·18 | 0·2734 |
| Min–max | 0·07–4·00 | 0·16–3·42 |  |
| **Doctor/clinic visits for consultation** |  |  |  |
| *Had ≥ 1 doctor visit, n (%)* |  |  |  |
| Yes | 19 (51·4) | 9 (24·3) |  |
| No | 17 (45·9) | 27 (73·0) |  |
| Unknown | 1 (2·7) | 1 (2·7) |  |
| *Number of visits per month (among patients with ≥ 1 visit)* |  |  |  |
| Mean (SD) | 0·82 (1·01) | 1·35 (0·98) |  |
| Median | 0·39 | 0·49 | 0·3594 |
| Min–max | 0·15–4·00 | 0·06–3·39 |  |

^a^Patients with inconsistent data values for resource use (*n* = 3) were excluded; ^b^*P* values based on Wilcoxon signed-rank test for paired data.
SD, standard deviation.
